# The complete mitochondrial genomes of *Gobiobotia meridionalis* (Cypriniformes: Cyprinidae)

**DOI:** 10.1080/23802359.2021.1917317

**Published:** 2021-04-26

**Authors:** Qin Ma, Lin Chen, Yuwen Tan, Yan Ren

**Affiliations:** a College of Life Science, Nanchang Normal University, Nanchang, PR China; b The Key Laboratory of Aquatic Biodiversity and Conservation, Institute of Hydrobiology, Chinese Academy of Sciences, Wuhan, PR China; c University of Chinese Academy of Sciences, Beijing, PR China; d Wuhan Academy of Agricultural Sciences, Wuhan, PR China

**Keywords:** *Gobiobotia meridionalis*, mitochondrial genome, phylogeny

## Abstract

In this study, we sequenced the complete mitogenome of *Gobiobotia meridionalis* (Chen et Tsao, 1982). The genome is 16,609 base pair (bp) in length, encoding 13 protein-coding genes (PCGs), 22 tRNA genes, 2 rRNA genes, and 1 non-coding control region (D-loop). The nucleotide composition is A: 30.34%, T: 26.88%, G: 16.49%, and C: 26.29% (AT content: 55.22%). The complete mitogenome of *G. meridionalis* provides essential and important DNA molecular data for the genetic diversity conservation of this species.

*Gobiobotia meridionalis* (Chen et Tsao, 1982), is a small-sized bottom-dwelling fish endemic to China, mainly distributed in Zhujiang basin, the tributaries of the middle Yangtze River, the Yuanjiang River, and the lower reach of the Lancang River (Chen 1998). It is easy to distinguish *G. meridionalis* from other species of genus *Gobiobotia*, for absence of the basal process of the pelvic fin bone (He 1997). Here, we first determined the complete mitochondrial genome of *G. meridionalis* and reconstructed the phylogenetic relationship with other Gobiobotinae species. It may shed light on some genetic background of *G. meridionalis*, and could provide essential and important DNA molecular data for the genetic diversity conservation of this species.

The specimen of *G. meridionalis* used in this study were obtained from the Fuhe River (116°14′10′′E, 26°33′03′′N), Jiangxi, China. Muscles were immediately fixed in 95% ethanol until it was picked out for DNA extraction. Some specimen of *G. meridionalis* was deposited in Nanchang Normal University (maqindoris@163.com) under the Voucher number NCNU20200828032.

Total genomic DNA was extracted from muscle samples of *G. meridionalis* using E.Z.N.A.^®^ Tissue DNA Kit (OMEGA,Guangzhou, China) following the manufacturer’s instructions and then sequenced on the Illumina HiSeq platform. The reads were assembled in NOVOPlasty version 4.0 (Dierckxsens et al. 2017,  https://github.com/ndierckx/NOVOPlasty) and annotated using MITOS webserver (Bernt et al. 2013). Protein-coding genes (PCGs) and rRNAs were rechecked by aligning them with the published mitogenomes of the *Gobiobotia filifer* (Garman, 1912) (Li et al. 2016).

The complete mitogenome of *G. meridionalis* was a circular DNA of 16,609 bp in length (GenBank with the accession number of MW442088) and contained 13 PCGs (*cyt* b, *ATP6*, *ATP8*, *COX1*-*3*, *ND1*-*6*, and *ND4L*), 22 tRNA genes, 2 rRNA genes (12S and 16S rRNA), and 1 control region (CR or D-Loop). It played similar patterns in gene arrangements, codon use, and gene overlaps, which have been reported in other Gobioninae mitogenomes (Hwang et al. 2013; Li et al. 2018). Eight tRNA genes (*Ala*, *Asn*, *Cys*, *Gln*, *Glu*, *Tyr*, *Ser*, and *Pro*) and NADH dehydrogenase subunit 6 (ND6) are encoded on the light strand (L-strand), the other 29 genes are encoded on the heavy strand (H-strand). The nucleotide composition is A: 30.34%, T: 26.88%, G: 16.49%, and C: 26.29% (AT content: 55.22%). Almost all 13 PCGs for *G. meridionalis* share the regular initiation codon ATG except *COI* gene with GTG. There are three different patterns of termination codons: nine PCGs (terminated with the stop codons TAA or TAG, while three PCGs (*cyt* b, *COX2*, *COX3*, and *ND4*) use incomplete stop codon (TA– or T–).

The phylogenetic trees of Gobiobotinae were reconstructed based on whole mitogenome dataset. Phylogenetic relationships of Gobiobotinae were reconstructed based on the multiple alignments of 17 mitochondrial genomes within this subfamily (Hwang et al. 2013; Hwang et al. 2014a, 2014b; Li et al. 2015, 2016, 2018; He et al. 2016; Tao and Zhao 2016). Outgroup taxa were selected based on a previous study (Tang et al. 2011). Neighbor-joining (NJ) analysis was conducted using MEGA version 7 (Kumar et al. 2016,  https://www.megasoftware.net) with 1000 bootstrap replicates. As in the previous study, our phylogeny also revealed that the genera *Gobiobotia* was monophyletic (Tang et al. 2011; Li et al. 2018). The phylogenetic tree strongly supported the close relationship of *Gobiobotia naktongensis* (Mori, 1935), *Gobiobotia pappenheimi* (Kreyenberg, 1911), *Gobiobotia intermedia* (Banarescu *et* Nalbant, 1968), *G. filifer*, and *G. meridionaliss* (Figure 1). In the tree, *G. naktongensis*, *G. pappenheimi*, *G. intermedia*, *and G. filifer* formed a clade sister to *G. meridionalis*, which was also congruent with the previous studies (Tang et al. 2011).

**Figure 1.**
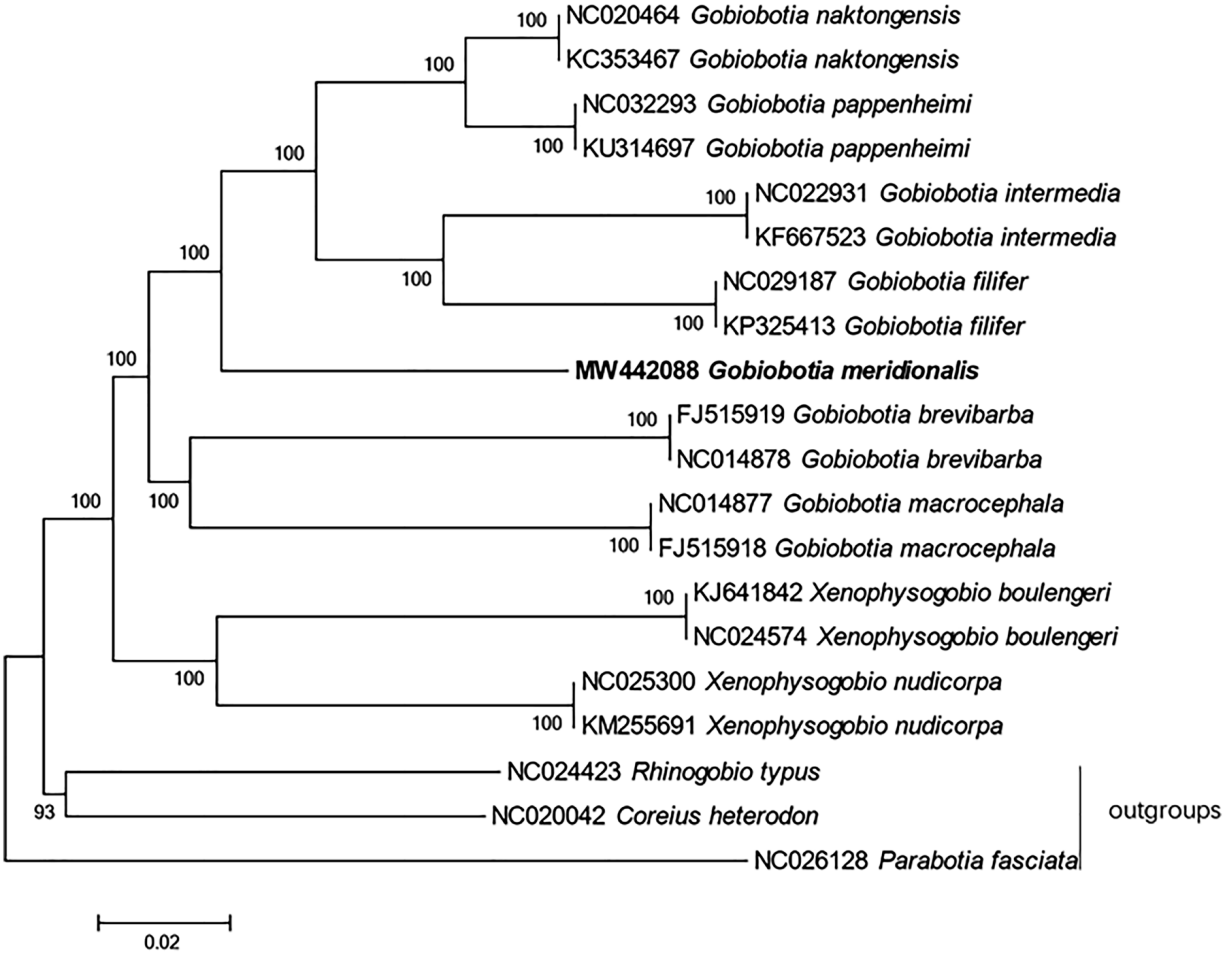
Phylogenetic tree of the subfamily Gobioninae using neighbor-joining (NJ) based on whole mitogenome sequences. Values at the nodes correspond to the support values for NJ methods.

## Data Availability

The data that support the findings of this study are openly available in Genbank with the accession codes MW442088 (https://www.ncbi.nlm.nih.gov/nuccore/MW442088).

## References

[CIT0001] Bernt M, Donath A, Juhling F, Externbrink F, Florentz C, Fritzsch G, Putz J, Middendorf M, Stadler PF. 2013. MITOS: improved de novo metazoan mitochondrial genome annotation. Mol Phylogenet Evol. 69(2):313–319. 22982435 10.1016/j.ympev.2012.08.023

[CIT0002] Chen YY. 1998. Fauna sinica, osteichthys, cypriniformes II. Beijing (China): Science Press..

[CIT0003] Dierckxsens N, Mardulyn P, Smits G. 2017. NOVOPlasty: de novo assembly of organelle genomes from whole genome data. Nucleic Acids Res. 45(4):e18. 28204566 10.1093/nar/gkw955 PMC5389512

[CIT0004] He B, Liu Y, Lai JS, Zhou J, Chen YY, Du J. 2016. The complete mitochondrial genome of *Xenophysogobio nudicorpa* (Teleostei, Cyprinidae, Gobiobotinae). Mitochondrial DNA Part A. 27(5):3322–1633. 10.3109/19401736.2014.958703 25231714

[CIT0005] He SP. 1997. On the anatomy and phylogeny of the Gobiobotine fishes (Cypriniformes: Cyprinidae). Acta Zootaxonom Sin. 16(4):490–495.

[CIT0006] Hwang DS, Byeon HK, Lee JS. 2013. Complete mitochondrial genome of the freshwater gudgeon, *Gobiobotia nakdongensis* (Cypriniformes, Gobioninae). Mitochondrial DNA. 24(4):409–410. 23387434 10.3109/19401736.2013.763247

[CIT0007] Hwang DS, Byeon HK, Lee JS. 2014a. Complete mitochondrial genome of the freshwater gudgeon, *Gobiobotia macrocephala* (Cypriniformes, Gobioninae). Mitochondrial DNA. 25(1):31–32. 23488918 10.3109/19401736.2013.775275

[CIT0008] Hwang DS, Byeon HK, Lee JS. 2014b. Complete mitochondrial genome of the freshwater gudgeon, *Gobiobotia brevibarba* (Cypriniformes; Gobioninae). Mitochondrial DNA. 25(1):33–34. 23527610 10.3109/19401736.2013.775276

[CIT0009] Kumar S, Stecher G, Tamura K. 2016. MEGA7: molecular evolutionary genetics analysis version 7.0 for bigger datasets. Mol Biol Evol. 33(7):1870–1874. 27004904 10.1093/molbev/msw054 PMC8210823

[CIT0010] Li BJ, Fu F, Feng JR, Jean CT, Lee CY, Han CC. 2015. Complete mitochondrial genome of *Gobiobotia intermedia* (Cypriniformes, Cyprinidae). Mitochondrial DNA. 26(5):803–804. 24409852 10.3109/19401736.2013.855901

[CIT0011] Li Q, Liu Y, Zhou J, Gong Q, Li H, Lai JS, Li LM. 2016. The complete mitochondrial genome of *Gobiobotia filifer* (Teleostei, Cypriniformes: Cyprinidae). Mitochondrial DNA Part A. 27(5):3325–3326. 10.3109/19401736.2015.1018205 25806579

[CIT0012] Li YH, Cao K, Fu CZ. 2018. Ten fish mitogenomes of the tribe Gobionini (Cypriniformes: Cyprinidae: Gobioninae). Mitochondrial DNA Part B. 3(2):803–804. 33490537 10.1080/23802359.2018.1467236 PMC7800260

[CIT0013] Tang KL, Agnew MK, Chen WJ, Hirt MV, Raley ME, Sado T, Schneider LM, Yang L, Bart HL, He SP, et al. 2011. Phylogeny of the gudgeons (Teleostei: Cyprinidae: Gobioninae. Mol Phylogenet Evol. 61(1):103–124. 21672635 10.1016/j.ympev.2011.05.022

[CIT0014] Tao WJ, Zhao HP. 2016. The complete mitogenome of *Xenophysogobio boulengeri* (Cypriniformes; Cyprinidae). Mitochondrial DNA Part A. 27(2):1283–1284. 10.3109/19401736.2014.945557 25133696

